# Characteristics of ecosystem multifunctionality and influencing factors of different grassland types in temperate desert of Longzhong Loess Plateau

**DOI:** 10.3389/fpls.2025.1619948

**Published:** 2025-07-17

**Authors:** Yali Li, Guoxing He, Xiaoni Liu, Tong Ji

**Affiliations:** ^1^ Key Laboratory of Grassland Ecosystem, Ministry of Education, Pratacultural College, Gansu Agricultural University, Lanzhou, Gansu, China; ^2^ Sino-U.S. Center for Grazing Land Ecosystem Sustainability, Lanzhou, Gansu, China

**Keywords:** grassland type, ecosystem multifunctionality, plant community characteristics, plant diversity, soil factors

## Abstract

**Introduction:**

Ecosystem multifunctionality (EMF) refers to the integrated capacity of an ecosystems to provide a variety of services and functions. It plays a crucial role in maintaining ecological balance and achieving sustainable grassland development. Grassland type is a classification unit based on dominant species within the grassland ecosystem. However, the response of EMF to grassland type is still unclear, and its influencing factors need to be studied in depth.

**Methods:**

In this study, we investigated the distribution characteristics of EMF in the Longzhong Loess Plateau temperate desert under different grassland types (*Kalidium foliatum* type, *Reaumuria songarica* type, *Salsola passerinum* type, and *Sympegma regelii* type grasslands) and identified the key factors driving changes in EMF.

**Results and discussion:**

The results indicated the *K. foliatum* type grassland exhibited higher EMF and belowground ecosystem multifunctionality (BEMF) indices (0.80 and 0.77, respectively), significantly greater compared to *R. soongorica* type, *S. passerine* type, and *S. regelii* type grasslands (*P*< 0.05). EMF and BEMF were significantly correlated with plant factors (coverage, root-shoot ratio), soil factors (mean weight diameter, MWD and geometric mean diameter, GMD) (*P<* 0.05), and showed strong positive relationships with Simpson diversity index. Hierarchical partitioning further indicated that MWD and GMD explained 29.86% and 38.21% of the variance in EMF, respectively. Structural equation modeling revealed that soil factors (MWD, GMD), plant factors (coverage, root-shoot ratio), and Simpson diversity index collectively explained 86% of the variation in EMF. Soil factors primarily exerted direct effects and indirectly promoted EMF by enhancing plant factors, with a total effect of 0.921. These findings suggest that *K. foliatum* type grassland can maintain EMF through soil factors, thereby supporting the sustainable development of temperate desert ecosystems in the Longzhong region of the Loess Plateau.

## 1 Introduction

Ecosystem multifunctionality (EMF) refers to the capacity of ecosystems to provide multiple services and functions (Yao et al., 2025). These functions such as net primary productivity, carbon sequestration, climate regulation, soil and water conservation, windbreaks and sand fixation, as well as species richness, among others (Hector and Bagchi, 2007). In recent years, global climate change and human activities have led to the degradation of grassland ecosystems, resulting in a decline in EMF (Li et al., 2024b; Yao et al., 2025). This decline disrupts ecosystem balance and poses a threat to the sustainable development of human societies (Bardgett et al., 2021; Wu et al., 2021). Against this background, scholars have begun to focus on the synergistic variations of functions such as soil retention, biodiversity maintenance, and material cycling within grassland ecosystems under different plant community conditions, conducting systematic investigations (Cheng et al., 2021; Yao et al., 2025). However, current research on the influencing factors of EMF under different grassland types remains insufficient, and the mechanisms by which different plant compositions affect EMF have not been systematically elucidated. This gap in knowledge limits a deeper understanding of the relationship between environmental factors and EMF, thereby constraining the optimization of ecosystem management strategies. Therefore, conducting systematic investigations into the influencing factors of EMF under various grassland types is of great significance for the sustainable utilization and conservation of grassland ecosystems.

As the fundamental units of grassland classification, grassland types are formed by different dominant plant species under similar habitat conditions and utilization modes (Li et al., 2024c). The species assembly, density, age, and growth stage of dominant plants vary among different grassland types, which significantly influences the plant community structure within each types (Bezemer et al., 2006). These factors, in turn, affect grassland EMF (Huang et al., 2024; Shu et al., 2023). Relevant studies have indicated that EMF varies significantly due to differences in dominant plant species assembly (Li et al., 2024a). Specifically, changes in the composition of dominant plants among different grassland types can regulate plant productivity and carbon sequestration capacities, thereby impacting ecosystem stability and functionality, ultimately influencing EMF (Shu et al., 2023; Wang et al., 2020; Novák et al., 2020). Therefore, understanding the effects of plant characteristics in different grassland types on EMF is crucial for the scientific conservation and sustainable management of grassland resources.

Soil, as a critical component of the ecosystem, directly influences EMF through its physicochemical properties. The dominant plant species in different grassland types can affect soil nutrient content and structure (Li et al., 2024c). Studies have shown that soil factors, such as pH and soil structure, play a more prominent role in regulating EMF (Han et al., 2021). Moreover, degradation of soil structure, such as erosion or compaction, can lead to nutrient loss and weaken ecosystem functions (Xiong et al., 2023). Therefore, an in-depth investigation into the regulatory mechanisms of soil factors across various grassland types would help enhance ecosystem stability and multifunctionality, providing scientific support for regional ecological restoration.

The Longzhong Loess Plateau is located in the arid and semi-arid regions of northern China, serves as an important ecological security barrier in northwest China (Li et al., 2024b). The area features complex topography, fragile ecological environments, and has been significantly affected by overexploitation and climate change (Liu et al., 2022). Due to environmental degradation, indicators such as vegetation coverage, plant diversity, and soil nutrients have declined, posing serious threats to ecosystem structure and function (Sun et al., 2015; Zhou et al., 2025; Jiang et al., 2016; Gang et al., 2018). As the main grassland type of the Longzhong Loess Plateau, temperate deserts are influenced by soil structural heterogeneity and climate variability, resulting in the formation of distinct grassland types dominated by *Kalidium foliatum*, *Reaumuria songarica*, *Salsola passerinum*, and *Sympegma regelii* (Li et al., 2024c; Xie et al., 2024). Accordingly, we used four grassland types (*K. foliatum* type, *R. songarica* type, *S. passerinum* type, and *S. regelii* type) in the Longzhong Loess Plateau temperate desert to assess the differences in EMF among different grassland types and to explore the main driving factors influencing EMF. Our hypotheses were that 1) EMF would be higher in *K. foliatum* type grasslands compared to the other three types. This is based on its root system adaptations to drought and saline-alkaline environments, which can improve soil structure and thereby enhance ecosystem functions (Li et al., 2024c). And 2) soil factors would have a more significant effect on EMF. By conducting this study, we hope to deepen our understanding of regulatory mechanisms of EMF in temperate desert grasslands and to provide a scientific basis for ecological conservation and grassland management.

## 2 Materials and methods

### 2.1 Study site

The research area is situated in the Loess Plateau of central Gansu Province (32°11′–42°57′N, 92°13′–108°46′E), administratively spanning Lanzhou, Dingxi, Baiyin, and Linxia Prefecture. Encompassing approximately 4.26×10^5^ km^2^. Elevation ranges from 1300 to 4200 m. Annual precipitation is 240 mm, with a maximum daily rainfall of 40 mm. The absolute minimum temperature is -17.1°C, the maximum temperature is 33.2°C, the average annual temperature is 9°C, and the annual growing degree days amount to 3100°C. Soil dominated by sierozem soil (pH ≈7.7). The vegetation composition is predominantly grassland ecosystems, major plant species include *K. foliatum*, *R. songarica*, *S. passerinum* and *S. regelii* (Li et al., 2024b, 2024d).

### 2.2 Sample plot design

Grassland type data were obtained from the 1:1,000,000 Grassland Resource Map of China (available via the China Grassland and Ecological Network: http://www.ecograss.com.cn/). August 2021, four representative grassland types in the temperate desert zone of the Longzhong Loess Plateau were selected as experimental sites (**Supplementary Figure S1**):

1. *K. foliatum* type grassland (Kf): monodominant community with *K. foliatum*;
2. *R. soongorica* type grassland (Rs): characterized by *R. soongorica* as the keystone species;
3. *S. passerina* type grassland (Sp): featuring *S. passerina* as the predominant vegetation;
4. *S. regelii* type grassland (Sr): dominated by *S. regelii* communities.

**Figure 1:**
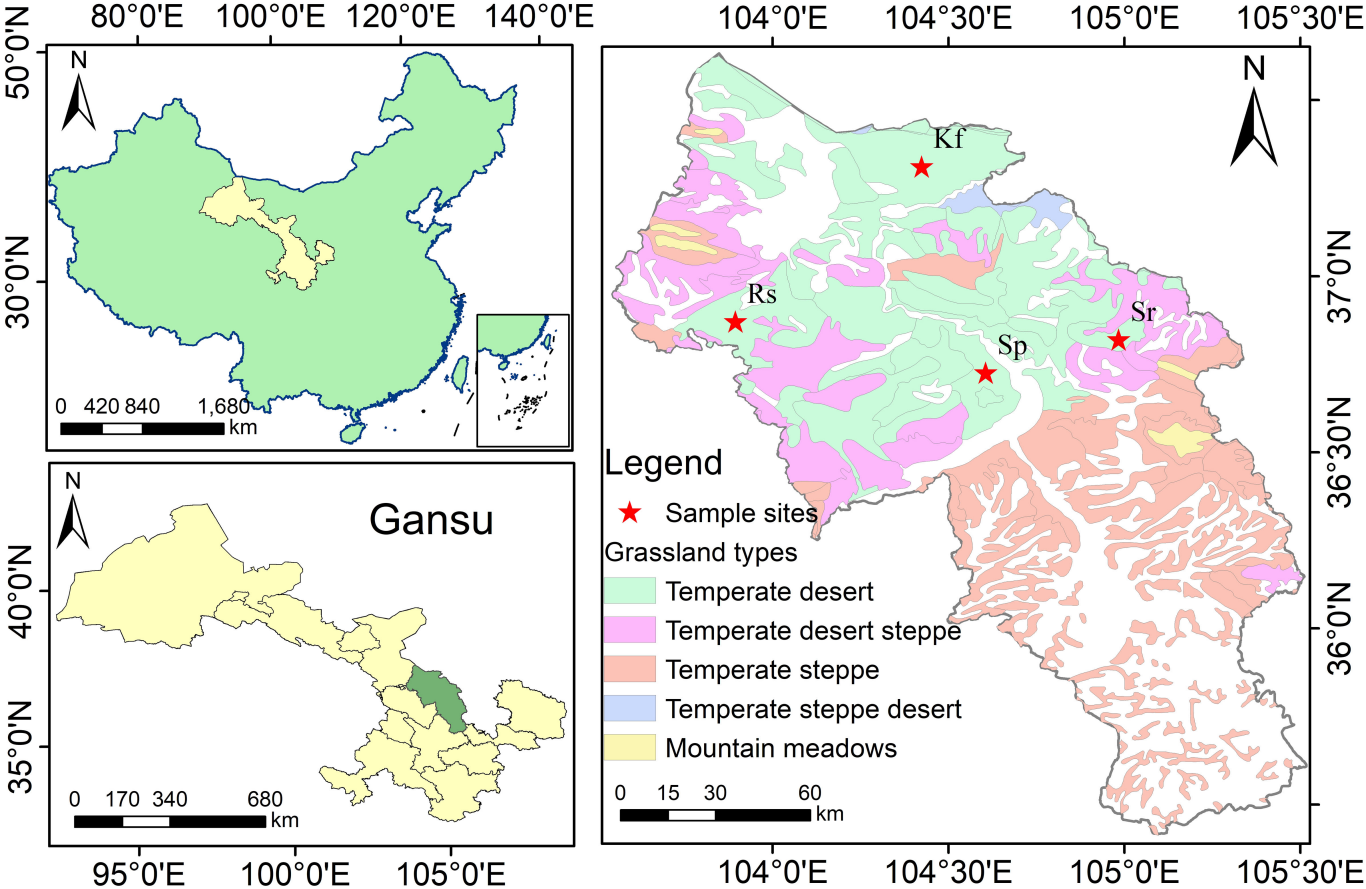
Distribution of sample. Kf, *K. foliatum* type grassland; Rs, *R.soongorica* type grassland; Sp, *S. passerina* type grassland; Sr, *S. regelii* type grassland.

Geospatial distributions and detailed site characteristics are illustrated in **Figure 1**. These sites were chosen based on their ecological representativeness and distinct dominant species assembly within the regional desert grassland biome. The area of each plot is 100 hm^2^. In each type of grassland plot, three independent sampling areas of 200 m × 200 m were established according to the principle of terrain homogeneity, with a spacing of ≥ 500 m between the sampling areas to avoid spatial interference.

### 2.3 Sample collection

#### 2.3.1 Vegetation sampling

In each sampling area, one randomly positioned 20 m × 5 m shrub quadrats were established. Within each shrub quadrat, three 1 m × 1 m herbaceous subplots were randomly delineated for vegetation surveys (**Supplementary Figure S2**). Shrub communities were assessed for species composition, height, canopy diameter, litter and aboveground biomass within the 20 m × 5 m quadrats. Herbaceous communities were characterized by species composition, height, coverage, and aboveground biomass in the 1 m × 1 m subplots. Total vegetation coverage and biomass per quadrat were recorded. Total biomass was derived by summing the biomass of the shrub layer and herbaceous layer.

#### 2.3.2 Soil physical properties sampling

Soil samples were collected within a 1 m × 1 m sampling plot after harvesting aboveground biomass. Using a stainless steel ring knife (volume: 100 cm^3^) and aluminum boxes, samples were taken at three depth intervals: 0–10 cm, 10–20 cm, and 20–30 cm. Three replicate samples were collected for each depth layer. Immediately after collection, the samples were sealed and transported to the laboratory for analysis of soil water content (using the 105°C drying method) and bulk density (using the ring knife method).

#### 2.3.3 Chemical properties sampling

Soil samples were collected using a soil auger with a diameter of 5 cm, following an “S” shaped five-point sampling method. Samples were vertically extracted from three depth intervals: 0–10 cm, 10–20 cm, and 20–30 cm. Each depth layer consisted of five borehole samples, which were combined into one composite sample (*n* = 3 composite samples per layer). The samples were placed in resealable bags and stored in the dark. After air-drying in the laboratory, roots (>2 mm) and gravel were removed, and the soil was ground through a 2 mm nylon sieve. The prepared samples were stored in a desiccator until chemical analysis, which included measurements of pH, organic matter, total nitrogen, and other relevant properties.

#### 2.3.4 Undisturbed soil sampling

Undisturbed soil columns (approximately 2 kg per layer) were collected using a flat-edged iron spade at three depth intervals: 0–10 cm, 10–20 cm, and 20–30 cm. The samples were placed in rigid plastic containers with cushioning pads and transported horizontally to minimize mechanical vibrations. After air-drying in the laboratory, the soil was manually crushed into 1 cm diameter fragments along natural fissures. These fragments were used for determining the composition of water-stable aggregates through wet sieving, with particle size classifications of >2 mm, 0.25–2 mm, 0.053 - 0.25 mm, and< 0.053 mm (Li et al., 2024c).

### 2.4 Vegetation characteristics

#### 2.4.1 Coverage, height, and aboveground biomass measurement

Coverage (%) was quantified using a grid-based visual estimation method. Grass layer height (cm) was measured vertically from the ground surface to the apex of undisturbed foliage using a ruler. Shrub aboveground biomass (g·m^-2^) was determined via the standard branch method and placed in labeled bags for measurement. Herbaceous aboveground biomass was harvested at ground level, sorted by species, and processed similarly. All samples were dried in a 65°C oven until reaching a constant weight (approximately 48 hours) before weighing. Total aboveground biomass was calculated as the sum of the herbaceous biomass and shrub biomass per unit area (g·m^2^).

#### 2.4.2 Belowground biomass measurement

Representative individual shrubs were selected, and the entire root system was excavated using a complete root extraction method. After removing the attached soil, the roots were placed in a 65°C oven to dry until reaching a constant weight. The dried root biomass was then weighed, and the biomass per unit area was calculated (g·m^2^). The belowground biomass was then obtained.

#### 2.4.3 Litter biomass measurement

Within the study area, litter samples were systematically collected from 1 m × 1 m plots after plant collection in August 2021. During each sampling event, all undecomposed litter within the plot was manually collected using sterile polyethylene gloves. The collected samples were immediately transported to the laboratory for processing. After carefully removing any foreign materials, the litter samples were dried at 65°C until reaching a constant mass (≥48 hours). Subsequently, the weight of the dried samples was measured using a precision balance, accurate to 0.01 grams. The litter biomass was then obtained.

#### 2.4.4 Leaf water content and root - shoot ratio

Leaves were collected from the selected shrubs and transported to the laboratory. After drying at 65°C for 48 hours, the water content of the leaves was measured.

The leaf water content (LWC) was calculated using the gravimetric method, expressed as (Wang et al., 2021):


$$\text{LWC}=\left(\frac{\text{Fresh weight}\left(\text{FW}\right)-\text{Dry weight}\left(\text{DW}\right)}{\text{Fresh weight}\left(\text{FW}\right)}\right)\times 100\%$$

where FW represents the fresh mass of leaves measured immediately after sampling, and DW denotes the constant mass obtained after oven-drying at 65°C for 48 hr.

Root-Shoot ratio, an indicator of biomass allocation between belowground and aboveground organs, was determined using (Qi et al., 2019):


$$\text{Root-Shoot ratio}=\frac{\text{Belowground biomass}}{\text{Aboveground biomass}}$$

#### 2.4.5 Plant diversity assessment

Community-level plant diversity was evaluated using four diversity indices (Shannon - Wiener diversity index, H; Simpson diversity index, D; Margalef richness index, R; Pielou evenness index, E) (Peng et al., 2018; Yao et al., 2025). For the specific calculation formulas, please refer to the **Supplementary Materials** (**Supplementary Table S1**).

### 2.5 Soil physicochemical characterization

#### 2.5.1 Physical properties

Soil bulk density (BD, g cm^-3^) was determined using the core method with stainless steel cutting rings (100 cm^3^ volume) (He et al., 2024). Soil water content (SWC, %) was calculated following gravimetric analysis (Yinglan et al., 2019):


SWC=(Fresh weight -Dry weight)/Dry weight×100%

Aggregate stability analysis was performed through wet sieving methodology. Air-dried soils (50 g) were fractionated using a nested sieve assembly (2 mm, 0.25 mm, 0.053 mm) in a mechanical sieve shaker. The protocol included: (i) 3-min saturation at controlled water level, (ii) vertical oscillation (3–4 cm amplitude) at 30 cycles min^-1^ for 5 min. Four aggregate fractions were obtained: > 2 mm, 0.25–2 mm, 0.053 - 0.25 mm, and< 0.053 mm (Cambardella and Elliott, 1993; Zhang et al., 2019). Each fraction was oven-dried at 65°C to constant mass and weighed (± 0.001 g).

Aggregate stability indices were computed as (Dou et al., 2020; Zeraatpisheh et al., 2021):


$$\text{MWD}=\sum_{i=1}^{n}\left({\text{X}}_{i}{\text{W}}_{i}\right)\;\;\;\;$$

$$\text{GMD}=\text{exp}\left[\frac{\sum_{i=1}^{m}\left({\text{W}}_{i}\ln{\text{X}}_{i}\right)}{\sum_{i=1}^{n}{\text{W}}_{i}}\right]\;$$

where *X_i_* represents the mean diameter (mm) of the *i*th fraction, *W_i_* is the mass percentage (%), MWD denotes mean weight diameter (mm), and GMD indicates geometric mean diameter.

#### 2.5.2 Chemical properties

Soil pH was measured in 1:2.5 (w/v) soil-water suspension using a calibrated pH meter (Basic pH Meter PB-10, European). Electrical conductivity (EC, μS·cm^-1^) was determined in the same extract with a conductivity probe (Conductivity meter DDS-608, Sichuan, China).

The soil analysis methods include: Soil organic matter (SOM, g·kg^-1^) via potassium dichromate oxidation with heating (He et al., 2024). Soil total nitrogen (TN, g·kg^-1^) using an automatic carbon and nitrogen analyzer (Primacs SNC 100-IC-E; Sklar, Netherlands). Soil alkaline hydrolyzed nitrogen (AN, mg·kg^-1^) through alkaline diffusion. Soil total phosphorus (TP, g·kg^-1^) measured by acid digestion with molybdenum-blue spectrophotometry (Dual Beam UV-Vis Spectrophotometer TH-1901, Beijing Pulse Analytical General Instrument Co). Soil available phosphorus (AP, mg·kg^-1^) via NaHCO_3_ extraction with colorimetry. Soil total potassium (TK, g·kg^-1^) by NaOH fusion and flame photometry (Flame Photometer Model FP640, Shanghai). And soil available potassium (AK, mg·kg^-1^) by NH_4_OAc extraction with flame photometric quantification.

### 2.6 Ecosystem multifunctionality assessment

The ecosystem multifunctionality index (EMF) was quantified through systematic integration of 11 key functional indicators representing primary productivity and biogeochemical cycles (Hector and Bagchi, 2007; Maestre et al., 2012a). The selected indicators encompassed: aboveground biomass, belowground biomass, soil water content, bulk density, organic matter, total nitrogen, alkaline hydrolyzed nitrogen, total phosphorus, available phosphorus, total potassium, available potassium. The belowground ecosystem multifunctional index (BEMF) is calculated based on nine indicators: soil water content, bulk weight, organic matter, total nitrogen, alkaline hydrolyzed nitrogen, total phosphorus, available phosphorus content, total potassium and available potassium. All soil factors are represented as average values for the 0–30 cm depth. The measured indicators comprehensively consider primary productivity and soil nutrient cycling.

The EMF was calculated using the mean value method (Maestre et al., 2012a; Zhai et al., 2024; Yao et al., 2025).

In the first instance, data standardization was performed using min-max normalization (Soliveres et al., 2016; Delgado-Baquerizo et al., 2020):


$${\text{Y}}_{i}=\left({\text{X}}_{i}-{\text{X}}_{\min}\right)/\left({\text{X}}_{\max}-{\text{X}}_{\min}\right)$$

where Y*_i_* denotes the standardized value (0–1 range) of the *i*th indicator, X*_i_* represents the observed value, with X_*min*_ and X_*max*_ corresponding to the minimum and maximum values across all sampling plots, respectively.

The composite EMF index was calculated as:


$$\text{EMF}=\sum_{i=1}^{N}{\text{Y}}_{i}/\text{N}$$

where N represents the number of standardized indicators. This additive approach ensures equal weighting across all functions following established protocols.

### 2.7 Statistical analysis

**Figure 2:**
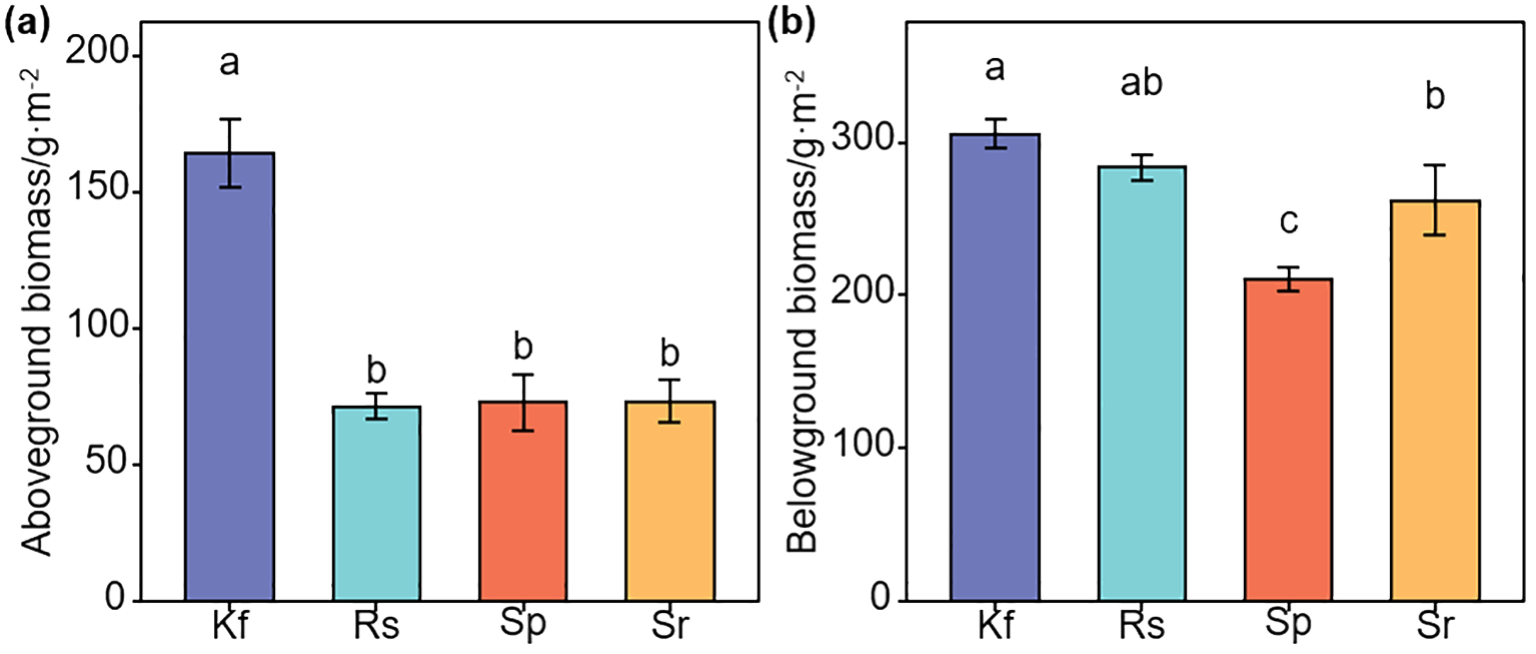
Effects of different grassland types on aboveground **(a)** and belowground **(b)** biomasses. Different lowercase letters indicate significant differences among the four grassland types (*P*< 0.05). Kf, *K. foliatum* type grassland; Rs, *R. soongorica* type grassland; Sp, *S. passerina* type grassland; Sr, *S. regelii* type grassland.

**Figure 3:**
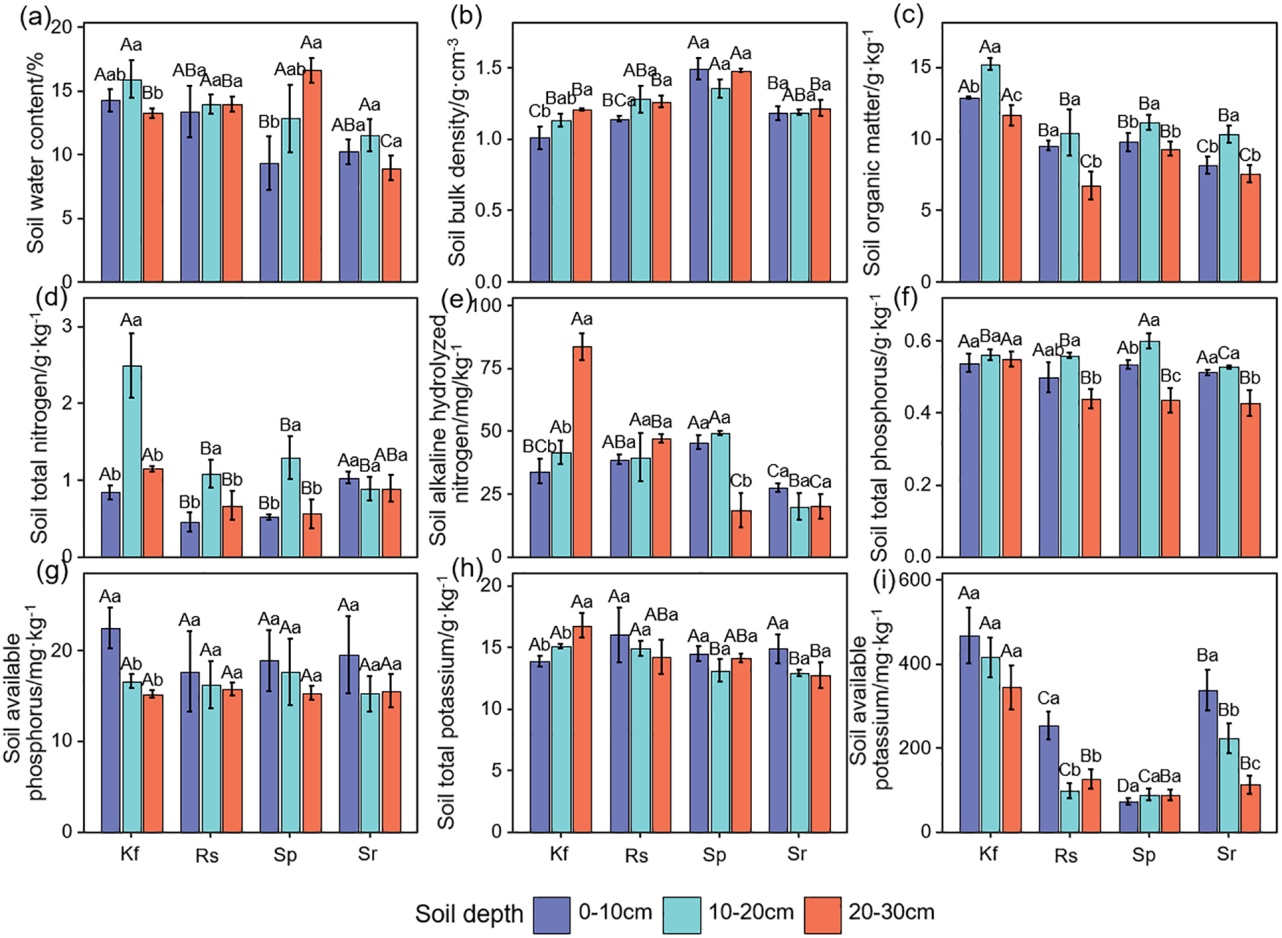
Effects of different grassland types on soil water content **(a)**, bulk density **(b)**, organic matter **(c)**, total nitrogen **(d)**, alkaline hydrolyzed nitrogen **(e)**, total phosphorus **(f)**, available phosphorus **(g)**, total potassium **(h)**, and available potassium **(i)**. Different uppercase letters indicate significant differences among the four grassland types (*P*< 0.05); different lowercase letters indicate significant differences among different soil layers within the same grassland type (*P*< 0.05). Kf, *K. foliatum* type grassland; Rs, *R. soongorica* type grassland; Sp, *S. passerina* type grassland; Sr, *S. regelii* type grassland.

**Figure 4:**
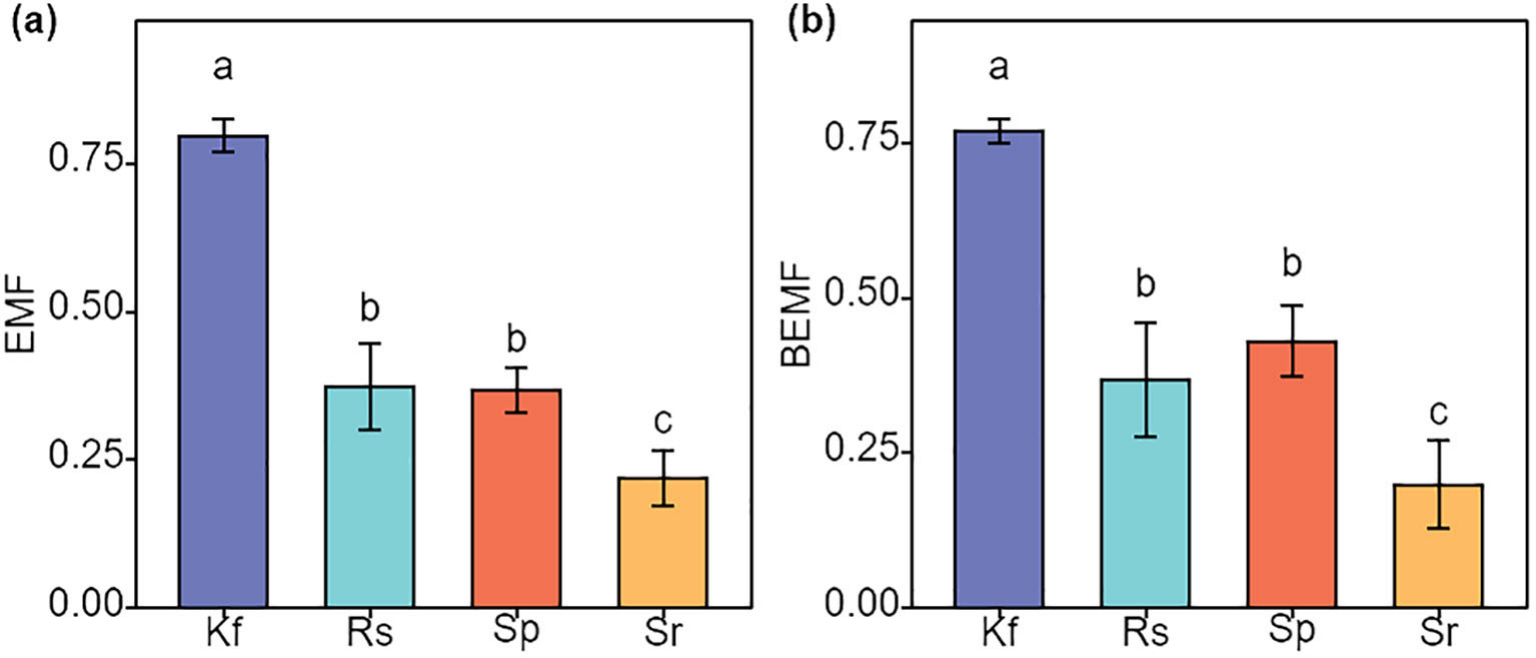
Effects of different grassland types on EMF, ecosystem multifunctionality **(a)**, and BEMF, belowground ecosystem multifunctionality **(b)**. Different lowercase letters indicate significant differences among the four grassland types (*P*< 0.05). Kf, *K. foliatum* type grassland; Rs, *R. soongorica* type grassland; Sp, *S. passerina* type grassland; Sr, *S. regelii* type grassland.

The data were initially processed using Excel 2010. Normality and homogeneity of variance were used the shapiro.test and bartlett.test functions from the R “Stats” package. Subsequently, one-way ANOVA and Least Significant Difference (LSD) tests were performed using the aov function from the “Stats” package to evaluate the differences in vegetation and ecosystem functioning indicators among different grassland types. Statistical significance was set at *P*< 0.05 (**Figures 2**–**4**; **Supplementary Figure S3**).

**Figure 5:**
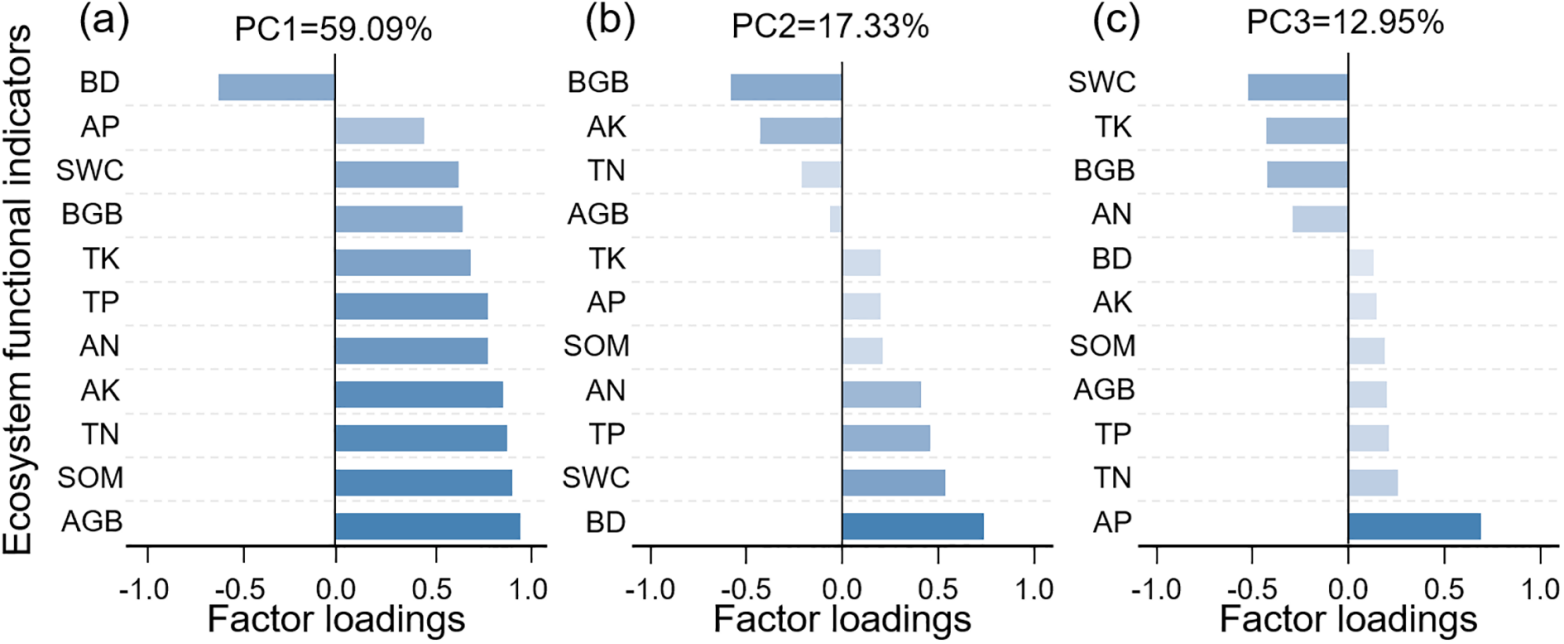
Relative contributions of single ecosystem functions to EMF. EMF: PC1 **(a)**, PC2 **(b)**, and PC3 **(c)**, ecosystem multifunctionality; AGB, Aboveground biomass; BGB, Belowground biomass; SWC, Soil water content; BD, Soil bulk density; SOM, Soil organic matter; TN, Soil total nitrogen; AN, Soil alkaline hydrolyzed nitrogen; TP, Soil total phosphorus; AP, Soil available phosphorus; TK, Soil total potassium; AK, Soil available potassium.

In this study, principal component analysis (PCA) was performed to reduce the dimensionality of the ecosystem function indicators. Three principal components with eigenvalues greater than 1 were extracted using the “FactoMineR” package (**Figure 5**). The contribution weights of each indicator to the principal components were calculated to assess their relative contributions to the EMF (Xiao et al., 2024).

**Figure 6:**
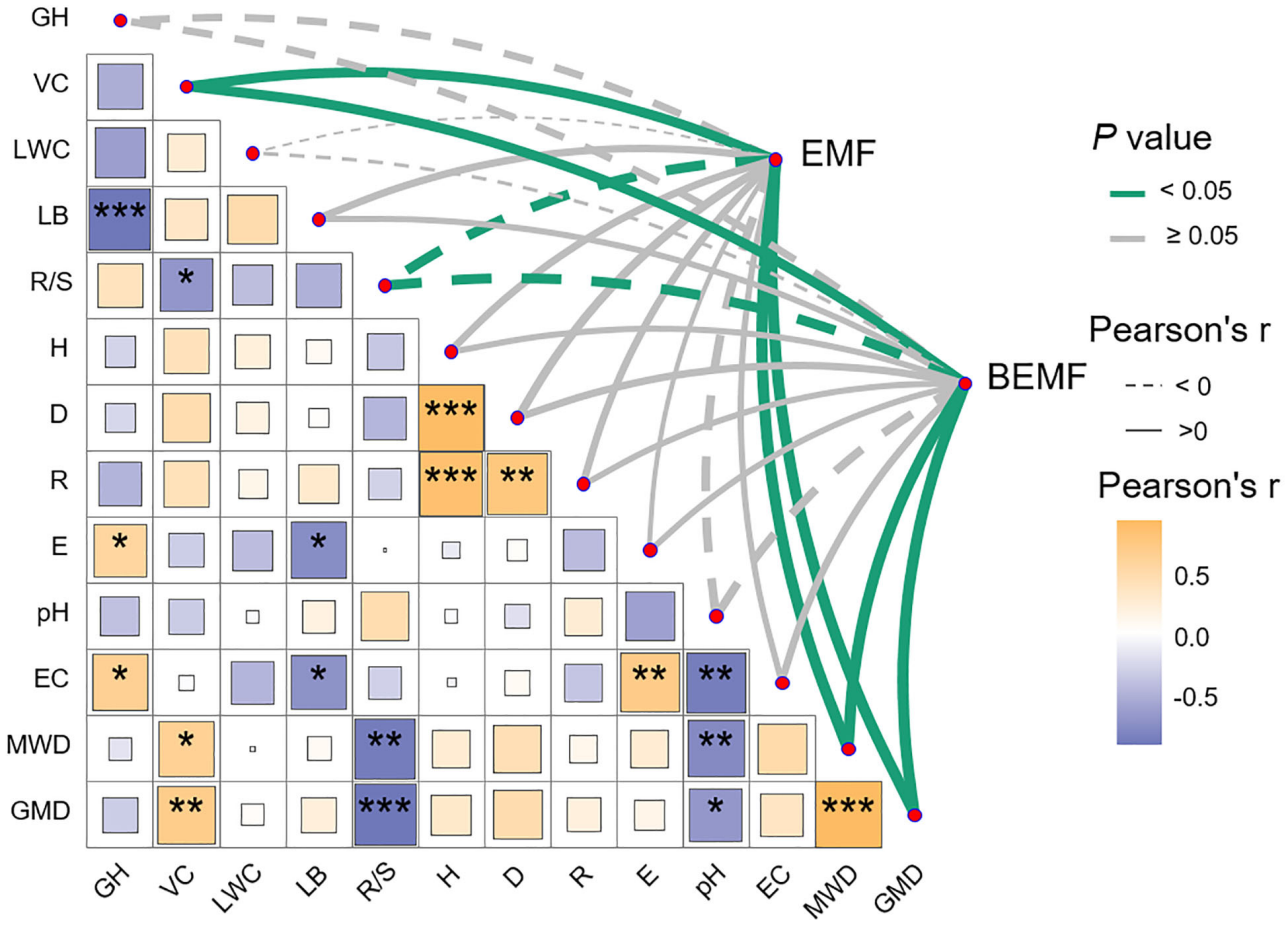
Pearson correlation analysis of ecosystem multifunctionality (EMF, BEMF), and plant and soil factors. EMF, Ecosystem multifunctionality; BEMF, Belowground ecosystem multifunctionality; GH, Height; VC, Coverage; LWC, Leaf water content; LB, Litter bionmass; R/S, Root-Shoot ratio; H, Shannon - Wiener index; D, Simpson index; R, Margalef richness index; E, Pielou evenness index; pH, Soil pH; EC, Soil electrical conductivity; MWD, Mean weight diameter; GMD, Geometric mean diameter. *p< 0.05; **p< 0.01; ***p< 0.001.

**Figure 7:**
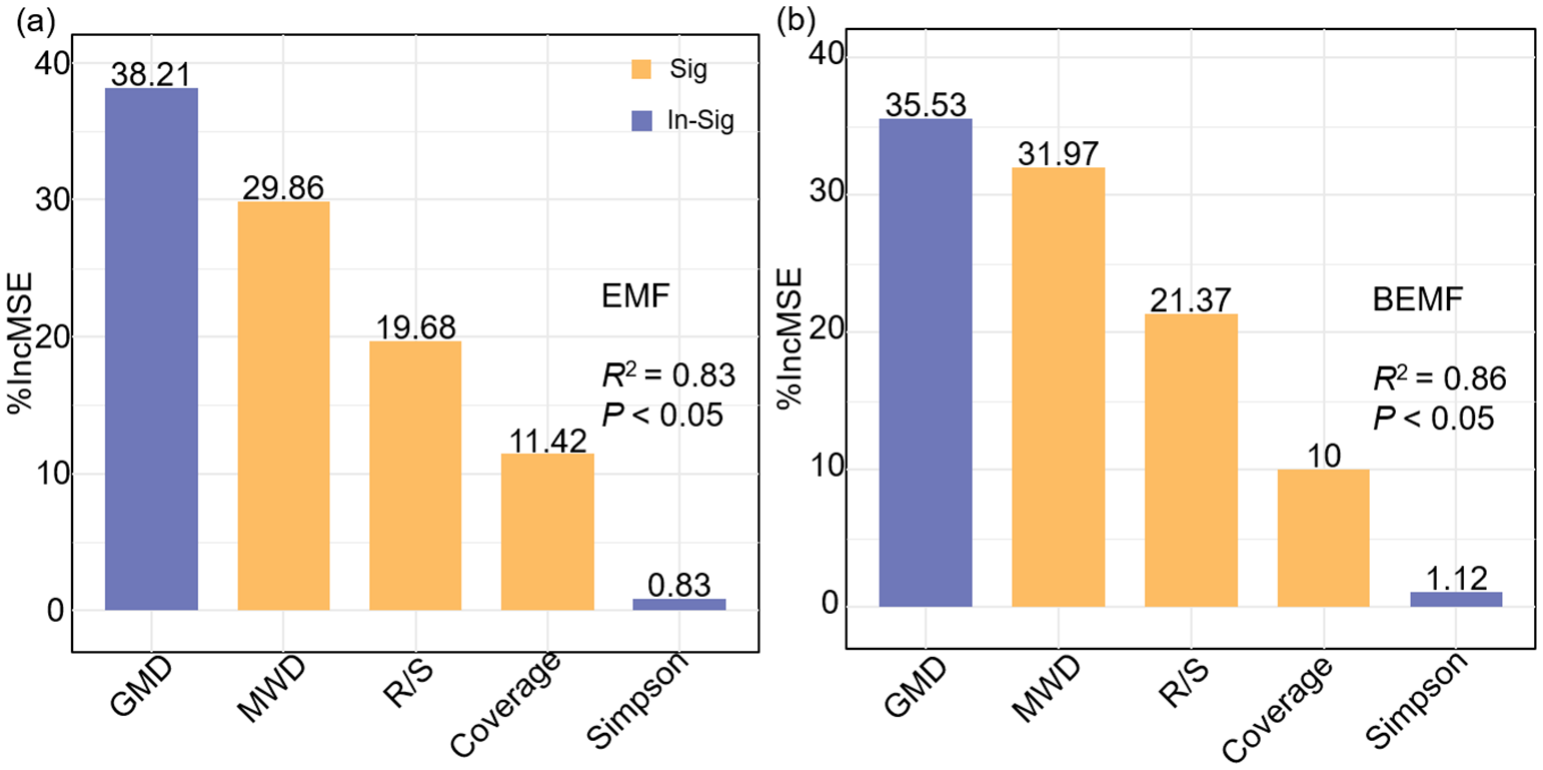
Importance of plant community characteristics and soil properties in EMF **(a)**, and BEMF **(b)**. EMF, Ecosystem multifunctionality; BEMF, Belowground ecosystem multifunctionality; R/S, Root-Shoot ratio; MWD, Mean weight diameter; GMD, Geometric mean diameter.

**Figure 8:**
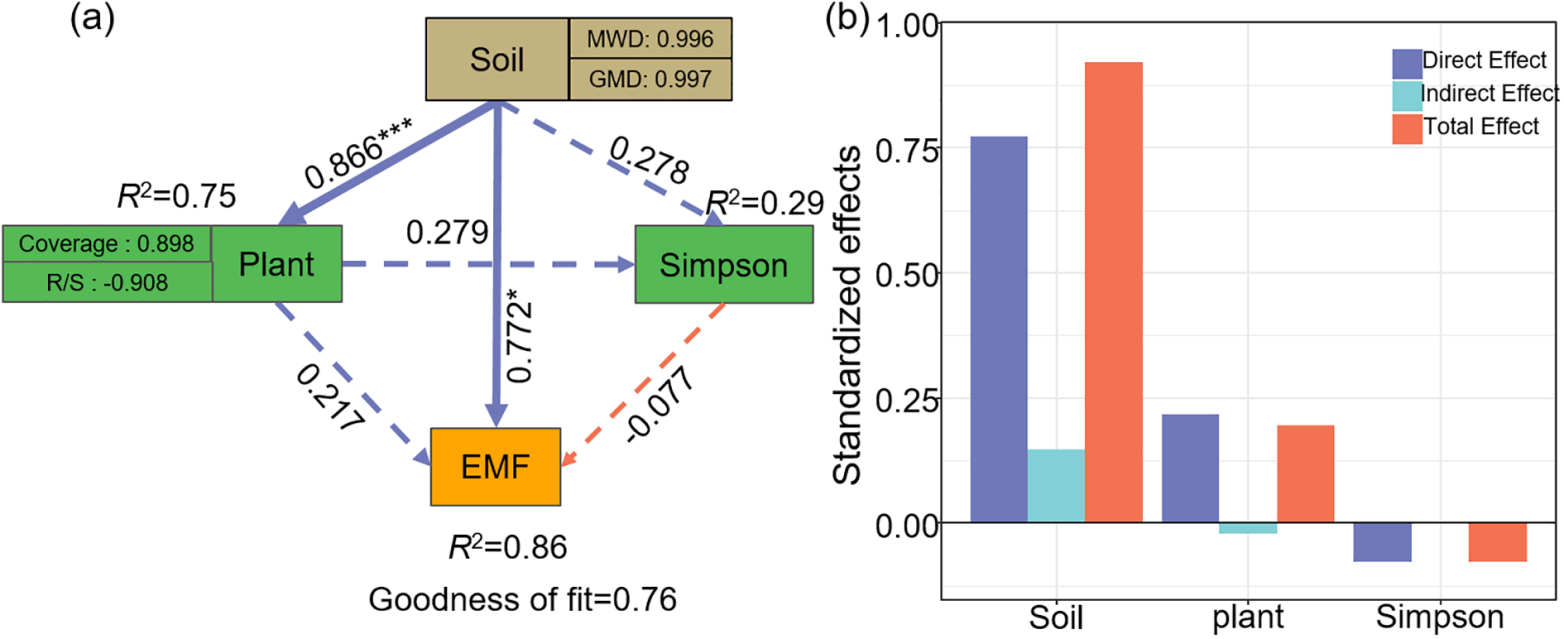
Effect of soil (MWD, GMD), plant (Coverage, Root-Shoot ratio), Simpson on EMF **(a)**; Standardized effects of driving factors on EMF **(b)**. Blue and orange arrows indicate positive and negative paths, respectively. Solid lines represent significant relationships, while dashed lines indicate insignificant relationships (*p< 0.05; ***p< 0.001). EMF, Ecosystem multifunctionality; R/S, Root-Shoot ratio; MWD, Mean weight diameter; GMD, Geometric mean diameter.

Pearson correlation analysis was conducted using the “Hmisc” package to evaluate the relationships among ecosystem multifunctionality, plant community characteristics, soil properties, and ecosystem functional indicators (**Figures 6**; **Supplementary Figure S4**). To further investigate the data, hierarchical partitioning analysis using the “glmm.hp” package was performed to identify the key factors influencing the changes in EMF and BEMF (**Figure 7**) (Liu et al., 2025). Finally, a Partial Least Squares Path Model (PLS-PM) was utilized to explore the direct and indirect effects of soil factors, plant factors, and plant diversity index on EMF (**Figure 8**), based on a priori model (**Supplementary Figure S5**). The PLS-PM model was implemented using the R package “plspm” (He et al., 2025). Figures were produced using R version 4.3.2 and ArcGIS 10.2 software (**Figure 1**).

## 3 Results

### 3.1 Differences in plant community characteristics between grassland types

Plant community characteristics varied among grassland types. *R. songarica* and *S. passerinum* type grasslands had taller plants (*P*< 0.05, **Supplementary Figure S3a**), while coverage was highest in *K. foliatum* type grassland (*P* > 0.05; **Supplementary Figure S3b**). *S. regelii* type grassland had the highest leaf water content, and *R. songarica* type grassland the lowest (*P*< 0.05; **Supplementary Figure S3c**). Biomass was greatest in *K. foliatum* type grassland (**Figures 2a, b**; **Supplementary Figure S3d**), with root-shoot ratios highest in *R. songarica* type grassland (*P*< 0.05, **Supplementary Figure S3e**). Plant diversity indices showed the highest values in *K. foliatum* type grassland (**Supplementary Figures S3f–h**). *S. passerinum* type grassland had highest Pielou evenness index (**Supplementary Figure S3i**).

### 3.2 Differences in ecosystem functional indicators of different grassland types

As can be seen in **Figure 3a**, the soil water content was highest in the *K. foliatum* type grassland in both the 0–10 and 10–20 cm soil layers, while it was highest in the 20–30 cm in the *S. passerina* type grassland (*P*< 0.05). With the deepening of the soil layer, the soil water content of the *K. foliatum* type, *R. soongorica* type and *S. regelii* type grasslands showed a tendency of first increasing and then decreasing, and reached the maximum at 10–20 cm, while the *S. passerina* type grassland showed a tendency of gradually increasing. *K. foliatum* type grassland had the lowest soil bulk density in all soil horizons and was significantly lower (*P*< 0.05) than *S. passerina* type grassland, but not significantly different from *R. soongorica* type and *S. regelii* type grasslands (*P* > 0.05, **Figure 3b**). In the vertical direction of the soil layer, the soil bulk density of both *K. foliatum* type and *S. regelii* type grasslands showed a slow increasing trend with the depth of the soil layer.

There were significant differences in soil organic matter content between grassland types (**Figure 3c**). And the soil organic matter content showed a tendency of first increasing and then decreasing with the deepening of the soil layer. Soil organic matter content was highest (*P*< 0.05) in the *K. foliatum* type grassland in the 0–30 cm layer and lowest (*P*< 0.05) in the *S. regelii* type grassland.

Soil total nitrogen, alkaline hydrolyzed nitrogen, total phosphorus, available phosphorus, total potassium, and available potassium were overall highest in the *K. foliatum* type grassland for all soil horizons (**Figures 3d–i**). There was no significant difference in soil available phosphorus content among different grassland types (*P* > 0.05), and the rest of the ecosystem function indicators were significantly different (*P*< 0.05). With the deepening of the soil layer, the soil total nitrogen and total phosphorus content in each grassland type showed a trend of first increasing and then decreasing. Soil alkaline hydrolyzed nitrogen content showed that the *K. foliatum* type and *R. soongorica* type grassland gradually increased with the deepening of the soil layer, *S. passerina* type grassland firstly increasing and then decreasing, and *S. regelii* type grassland gradually decreasing. Soil available phosphorus and available potassium content tended to decrease with deepening of the soil layer. Soil total potassium content of the *K. foliatum* type grassland showed a gradual increase with deepening of the soil layer, the *R. soongorica* type and *S. regelii* type grassland showed a gradual decrease, and the *S. passerina* type grassland showed a first decreasing and then an increasing trend.

### 3.3 Differences in ecosystem multifunctionality of different grassland types

The mean method was used to calculate EMF, and BEMF and to compare the differences in EMF, and BEMF between the different grassland types. As can be seen from **Figures 4a, b**, the *K. foliatum* type grassland had EMF, and BEMF indices at 0.80 and 0.77, respectively, which were significantly higher (*P*< 0. 05) than in the *R. soongorica* type, *S. passerina* type and *S. regelii* type grassland. The *R. soongorica* type grassland did not have significantly difference in EMF and BEMF compared to *S. passerina* type grassland (*P* > 0.05), but were significantly higher (*P*< 0. 05) than *S. regelii* type grassland.

Three common factors were extracted from the 11 indicator variables (**Figure 5**), accounting for variance contributions of 59.09%, 17.33%, and 12.95%, respectively, with a cumulative contribution rate of 89.37%. Among them, aboveground biomass, soil organic matter, total nitrogen, and available potassium predominantly load on Factor 1 (**Figure 5a**). Soil bulk density, belowground biomass, and soil water content were primarily associated with Factor 2 (**Figure 5b**); and soil available phosphorus and soil water content mainly load on Factor 3 (**Figure 5c**). Regarding the contribution of individual ecosystem function indicators to EMF, soil available potassium, aboveground biomass, total nitrogen, and soil organic matter have relatively higher proportions (**Figure 5**).

### 3.4 Influence of plant community characteristics and soil properties on ecosystem multifunctionality

According to the correlation analysis, aboveground biomass, soil organic matter, and total nitrogen were significantly positively correlated with EMF, BEMF, mean weight diameter, and geometric mean diameter (*P*< 0.05), while showing a significant negative correlation with the root-shoot ratio (**Supplementary Figure S4**). Correlation analysis revealed that both EMF and BEMF were significantly positively correlated with coverage, mean weight diameter, and geometric mean diameter, while showing a significant negative correlation with the root-shoot ratio (*P*< 0.05). Furthermore, they exhibited a strong positive correlation with the Simpson index (**Figure 6**).

Based on the previous analysis, we selected vegetation factors strongly associated with ecosystem multifunctionality (Coverage and Root-Shoot ratio), Simpson index, and soil factors (mean weight diameter and geometric mean diameter) for hierarchical partitioning and the construction of a structural equation model. The results indicated that the most important explanatory factors for EMF were geometric mean diameter (38.21%) and mean weight diameter (29.86%). These explanatory factors collectively explained 83% (*P*< 0.05) of the EMF variation, with the most highly explained of the explanatory factors being soil geometric mean diameter (**Figure 7a**). The results obtained from screening the explanatory factors of BEMF were found to be more similar to those obtained from the screening of EMF. Among them, the soil geometric mean diameter (35.53%) and mean weight diameter (31.97%) showed a higher degree of explanation compared to other indicators of BEMF (**Figure 7b**). From the results in different hierarchical partitioning analysis, soil factors (mean weight diameter and geometric mean diameter) can better explain the variation in EMF.

Structural equation modelling (SEM) analysis further indicated that soil factors (mean weight diameter and geometric mean diameter), plant factors (coverage and root-shoot ratio), and Simpson diversity index collectively explained 86% of the variation in EMF (*R* ^2^ = 0.86, **Figure 8a**). On the one hand, soil factors directly influenced EMF (standardized coefficient: β = 0.772), exhibiting a significant positive effect (*P*< 0.05). On the other hand, soil factors indirectly enhanced EMF by improving plant factors and Simpson diversity index. The total effects of the soil factors, plant factors, and Simpson diversity index on EMF were 0.921, 0.196, and -0.077, respectively (**Figure 8b**).

## 4 Discussion

### 4.1 Effects of different grassland types on plant community characteristics, ecological function indicators and ecosystem multifunctionality

This study selected four grassland types on the Loess Plateau (*K. foliatum* type, *R. songarica* type, *S. passerinum* type, and *S. regelii* type) to analyze plant community characteristics, ecological function indicators, and ecosystem multifunctionality. The study found that the height of plants in the research area ranged from 21 to 36 cm, and the vegetation coverage ranged from 28% to 36%. This indicates that the coverage in the temperate desert area is relatively low, which is consistent with the findings of Zhang and Zhao (2015) in the Hexi Corridor region of Gansu. Leaf water content reflects the drought resistance and environmental adaptability of desert plants (Wang et al., 2021). This study found that grassland types dominated by *R. songarica* exhibited the lowest leaf water content, while those dominated by *S. regelii* showed the highest. These findings are consistent with Li et al. (2020), who also reported that grasslands with *R. songarica* as the dominant species had lower leaf water content compared to other grassland types. The root - shoot ratio is used to evaluate the allocation characteristics of aboveground and belowground biomass in plants. A high root - shoot ratio indicates that plants allocate more resources to their root systems, while a low root - shoot ratio suggests that more resources are invested in the growth of the aboveground parts (Qi et al., 2019; Wang et al., 2023b). This study indicated that the root - shoot ratio is highest in the *R. songarica* type grassland, primarily because of the well-developed root systems of the dominant species, *R. songarica*, combined with a lower aboveground biomass (**Figure 2a**), which enables better adaptation to arid environments.

This study found that temperate desert grassland communities of different types exhibited relatively low biodiversity, with significant differences in diversity indices, although their overall variation patterns were largely consistent. The *K. foliatum* type grassland showed the highest values for the Shannon - Wiener, Simpson, and Margalef richness indices, whereas the community dominated by *S. regelii* exhibited the lowest values for all diversity indices. This is primarily because the grassland type dominated by *S. regelii* has a more homogeneous species assembly compared to other grassland types, with poor species distribution uniformity, resulting in a pronounced single-species community and poor performance in species diversity. The study by Dong et al. (2020) found that among eight typical shrub communities in the western section of the Hexi Corridor in Gansu, the diversity index of the *K. foliatum* community was the highest, while the vegetation communities had few species, relatively simple community structures, and very uneven species distribution, which is consistent with the results of this study. Osmonali et al. (2023) also found that the species assembly in desert plant communities in Kazakhstan is primarily dominated by a single dominant community. Additionally, the relatively single species assembly of the study area is closely related to the semi-arid climate of Longzhong. This has naturally led to the formation of a temperate desert community dominated by a single species, adapted to the arid and water-scarce habitat conditions (Li et al., 2024b). The grassland is primarily dominated by *K. foliatum*, *R. songarica*, *S. passerinum*, and *S. regelii* (Li et al., 2024c), which are minimally affected by external environmental influences, resulting in highly stable community structures. Moreover, temperate desert plants possess drought, cold, salt-alkali tolerance, and resilience to poor soils, enabling them to better adapt to the harsh, dry, and windy conditions with nutrient-deficient soils typical of Longzhong. In summary, the aboveground biomass of temperate desert vegetation in Longzhong is relatively low, while the belowground biomass accounts for a higher proportion. The litter biomass is the lowest, and overall vegetation coverage is low, resulting in relatively low plant diversity. The grassland dominated by *K. foliatum* generally exhibits higher ecological value. Thus, *K. foliatum* may play a positive role in the restoration of temperate desert vegetation.

This study analyzed the distribution characteristics of soil properties across different grassland types in the temperate desert of Longzhong. The results showed that the *K. foliatum* type grasslands had higher soil water content and lower bulk density, with bulk density increasing gradually with soil depth. This trend is attributed to the extensive root systems of *K. foliatum*, which primarily extend and intertwine within the surface soil, loosening the soil and thereby reducing the surface soil bulk density (Mueller et al., 2024). Significant differences in soil bulk density were observed among various grassland types within the same soil layer. This variation may be attributed to the different dominant plant species and their root distribution patterns across the grassland types, which consequently lead to differences in soil bulk density (Chen et al., 2024). Soil nutrients provide essential elements necessary for plant growth and development (Zeng et al., 2016; Li et al., 2024c). It is generally recognized that the primary sources of soil nutrients in grasslands mainly rely on plant residues and root secretions (Ochoa-Hueso et al., 2020). This study found that the overall nutrient content of soils in the temperate desert of the Longzhong region is relatively low, and the distribution characteristics among different soil layers and grassland types with different dominant plant species show a high consistency with previous research findings (Lu et al., 2023). Among them, the grassland type with *K. foliatum* as the dominant plant had the highest soil organic matter, total nitrogen and total phosphorus content. This is mainly due to the higher plant aboveground biomass, litter biomass and belowground biomass in the grassland type with *K. foliatum* as the dominant plant. Inputs of litter and root residues increase soil nutrient fixation in *K. foliatum* type grasslands (Li et al., 2021). At the same time, soil organic matter, total nitrogen and total phosphorus overall with the deepening of the soil layer showed a trend of first increasing and then decreasing, the highest in the 10–20 cm soil layer. This is due to the soil nutrients are mainly affected by the plant root system, the soil-forming parent material and other factors (Anderson, 1988). Together with the fact that the roots of the dominant plants of the temperate desert (*K. foliatum*, *R. songarica*, *S. passerinum*, and *S. regelii*) are mainly distributed in the sub-surface layer (10–20 cm), this results in a high level of nutrient content. Soil available nutrients, as key elements that plants can directly absorb, play a crucial role in plant growth and development (Li et al., 2024e). This study found that in the temperate desert of Longzhong, all four grassland types with *K. foliatum* as the dominant species exhibited higher levels of soil available nutrients. This is primarily attributed to the higher vegetation coverage, height, litter and belowground biomass, as well as elevated levels of total nutrients in these grasslands, which facilitate the accumulation of available nutrients and result in their higher content. In the vertical direction, the variation patterns of available phosphorus and available potassium are consistent, both showing a gradual decrease with increasing soil depth. This indicated that the temperate desert in Longzhong has a high efficiency in absorbing and utilizing nutrients from the soil surface layer. Conversely, the content of soil alkaline hydrolyzed nitrogen exhibits significant differences in its vertical distribution across different grassland types. These variations are influenced by factors such as vegetation coverage, dominant plant species, and plant characteristics.

The analysis of ecosystem multifunctionality indicated that the *K. foliatum* type grassland has the highest multifunctionality at both the overall and belowground ecosystem levels. The *R. songarica* type and *S. passerinum* type grasslands were ranked second, while the *S. regelii* type grassland has the lowest multifunctionality (**Figure 4**). This suggested that *K. foliatum* plants have a higher potential for restoring the multifunctionality of temperate desert ecosystems. The reasons for differences in ecosystem multifunctionality among the four grassland types may stem from significant variations in their soil physical and chemical properties. Such heterogeneity can influence key ecological processes, such as nutrient cycling and energy flow, thereby affecting the stability of the grassland ecosystem (Wang et al., 2023a). For example, the lower plant biomass in *S. regelii* type grasslands reduces the vegetation’s capacity to protect the soil.

### 4.2 Influence of plant and soil factors on ecosystem multifunctionality

Existing studies have identified plant community characteristics as the main factor regulating EMF (Xu et al., 2024; Yao et al., 2025). Among them, a significant positive correlation between plant diversity and EMF is now a widely shared view (Maestre et al., 2012b). Also studies on the Loess Plateau have shown that plant diversity can sometimes better explain EMF (Wang et al., 2022). This study also found that ecosystem multifunctionality showed an increasing trend with the rise in plant diversity indices (**Figure 6**). The relationship between plant diversity and EMF is altered by factors such as ecosystem type and disturbance factors (Dietrich et al., 2024). It has been demonstrated that the effect of species richness on EMF is dependent on the evenness of species in the community (Maestre et al., 2012b). In this study, plant root-shoot ratio showed a significant negative correlation with ecosystem multifunctionality, while vegetation coverage, mean weight diameter, and geometric mean diameter exhibited significant positive correlations. Additionally, the Simpson diversity index had a relatively strong positive correlation with ecosystem multifunctionality (**Figure 6**). It also showed that the plant community characteristics and soil properties influence ecosystem multifunctionality. It also showed that soil factors, such as mean weight diameter and geometric mean diameter, have a high explanatory rates for variations in ecosystem multifunctionality (**Figure 7**), indicating that soil aggregates play a key role in influencing ecosystem multifunctionality in the temperate desert regions of the Loess Plateau. Similar studies also found that soil conditions are primary factors determining ecosystem characteristics like plant community primary productivity (Van Breemen, 1993).

The results of the structural equation model indicated that environmental factors explain up to 86% of the variation in ecosystem multifunctionality (**Figure 8**). Soil factors, such as mean weight diameter and geometric mean diameter, have a direct positive effect on ecosystem multifunctionality. The productivity of arid and semi-arid ecosystems is significantly influenced by soil structure (Khasi et al., 2024). Han et al. (2021) found that soil aggregate structure is an important driver in shaping the grassland ecosystem multifunctionality. Our study also found that soil aggregates not only directly influence ecosystem multifunctionality, but also indirectly influence ecosystem multifunctionality by influencing plant factors and Simpson diversity index. This is mainly due to the fact that the mean weight diameter and geometric mean diameter, as indicators of soil aggregate stability, not only reflect the resistance of the soil structure to maintain its original form when exposed to external forces and environmental changes, but are also an important indicator for assessing the soil quality (Dou et al., 2020; Xia et al., 2022). Due to the low soil water content in the top layer of temperate deserts, there is a greater contribution to grassland nutrient content from the soil aggregate formation and stabilization of desert plants during their growth process (Zhang et al., 2024). This in turn affects plant growth and development, leading to the production of large amounts of plant residues, and this increased organic matter input affects plant diversity index, further affecting the grassland EMF (Wang et al., 2023a; Pichon et al., 2024). Improvements in soil structure may increase soil water and nutrient content, which may make it more sensitive to changes in EMF (Tian et al., 2024). In recent years, the temperate deserts of the Loess Plateau have become warmer and drier as a result of rising temperatures and decreasing precipitation (Miao et al., 2016). Although as reported in our study, *K. foliatum* desert plants favor the improvement of EMF indices, prolonged aridification in these areas may also greatly diminish their positive impact on ecosystem functions. Overall, *K. foliatum* desert plants can serve as a practical tool for enhancing ecosystem function in less arid grasslands, but it limits the ability of plant diversity to support multiple ecosystem functions. Our results provide valuable information for managing grasslands for biodiversity conservation.

## 5 Conclusion

Our study demonstrated that EMF, and BEMF showed the highest values in *K. foliatum* type grassland. Furthermore, soil factors (mean weight diameter, and geometric mean diameter) were more explanatory of EMF, suggesting that soil factors can more effectively predict EMF. Soil factors not only directly influence EMF, but may also indirectly drive changes in EMF through plant factors (coverage, root-shoot ratio) or Simpson diversity index. Therefore, in the process of ecological restoration, it should pay attention to the restoration of the interaction relationship between soil factors and plant factors. Meanwhile, *K. foliatum* plants have an important and positive role in achieving the restoration of desert grasslands on the Loess Plateau.

## Data Availability

The original contributions presented in the study are included in the article/**Supplementary Material**, further inquiries can be directed to the corresponding author.
