# Supplementary material for: Characteristics of ecosystem multifunctionality and influencing factors of different grassland types in temperate desert of Longzhong Loess Plateau
Source: Front Plant Sci. 2025 Jul 17;16:1619948. doi: 10.3389/fpls.2025.1619948 (PMC12310632; doi:10.3389/fpls.2025.1619948)
Supplement: Supplementary file 1 [file Table1.docx]

**Supplementary materials**

Characteristics of ecosystem multifunctionality and influencing factors of different grassland types in temperate desert of Longzhong Loess Plateau

Yali Li^1,2^, Guoxing He^1,2^, Xiaoni Liu^1,2^*, and Tong Ji^1,2^

^1^Key Laboratory of Grassland Ecosystem, Ministry of Education, Pratacultural College, Gansu Agricultural University, Lanzhou, Gansu, China, ^2^Sino-U.S. Center for Grazing Land Ecosystem Sustainability, Lanzhou, Gansu, China

*CORRESPONDENCE: Xiaoni Liu. E-mail: [liuxn@gsau.edu.cn](mailto:liuxn@gsau.edu.cn)


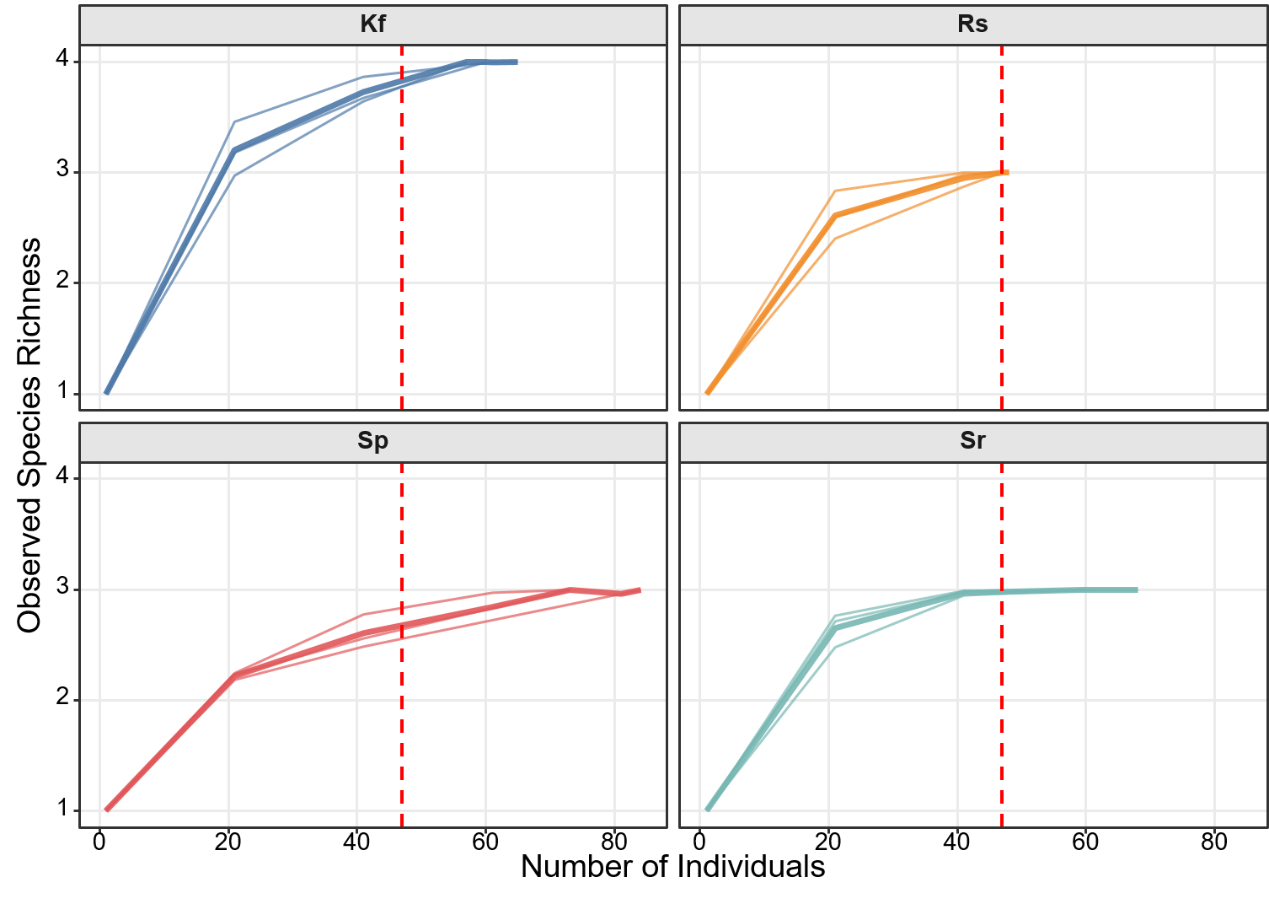


FIGURE S1 Rarefaction curves.


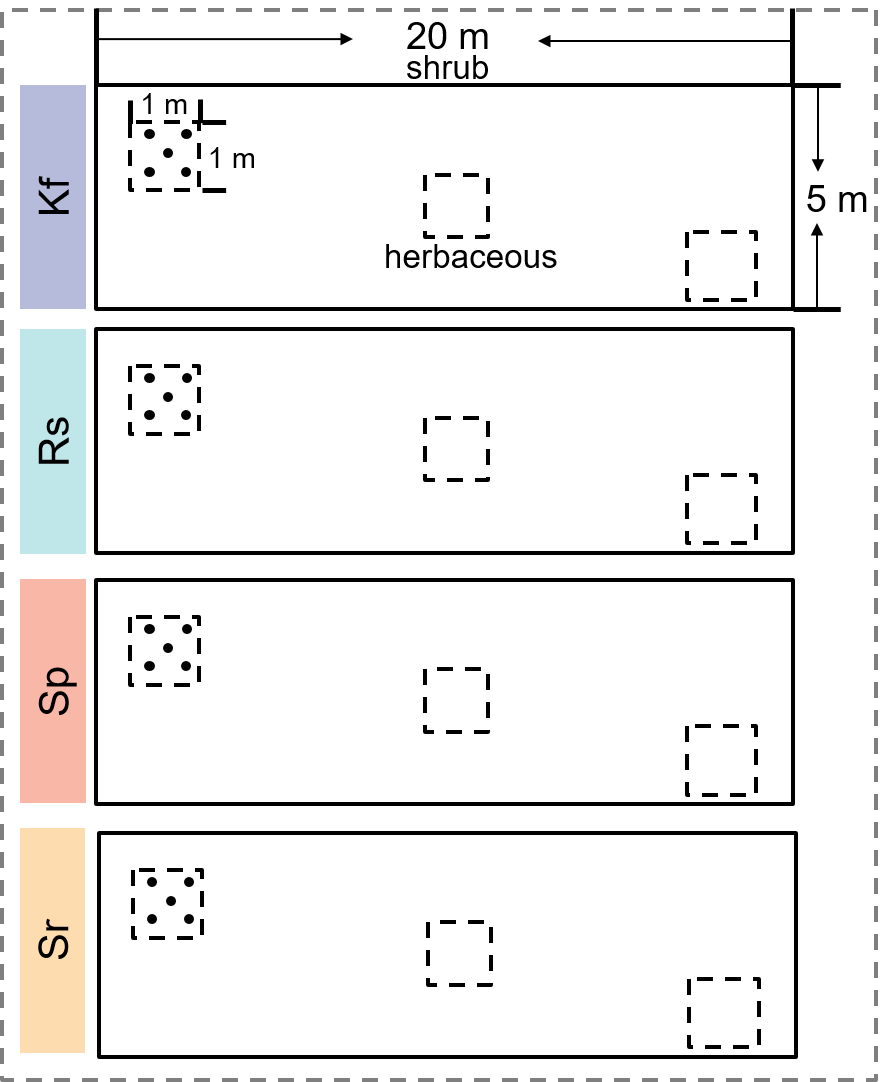


FIGURE S2 Sample plot sampling map.

TABLE S1 Plant diversity calculation formulas

| Plant diversity indices | Calculation formulas |
| --- | --- |
| Shannon - Wiener diversity index (H) | $\text{H=-}\sum_{\text{i}\text{=1}}^{\text{s}} \left( \text{P}_{\text{i}}\text{ln}\text{P}_{\text{i}} \right)$ |
| Simpson diversity index (D) | $\text{D=1-}\sum_{\text{i}\text{=1}}^{\text{s}} {\text{P}_{\text{i}}}^{\text{2}}$ |
| Margalef richness index (R) | $\text{R=}\left( \text{S-1} \right)/\text{lnN}$ |
| Pielou evenness index (E) | $\text{E=}\text{H}/\text{lnS}$ |
| Where: S = Total number of species in the quadrat, P*i* = Proportional importance value of species i relative to the total importance value, N = Total number of individual plants in the quadrat. Importance values(IV) were calculated as the average of relative height, relative coverage, and relative aboveground biomass for each species. | |


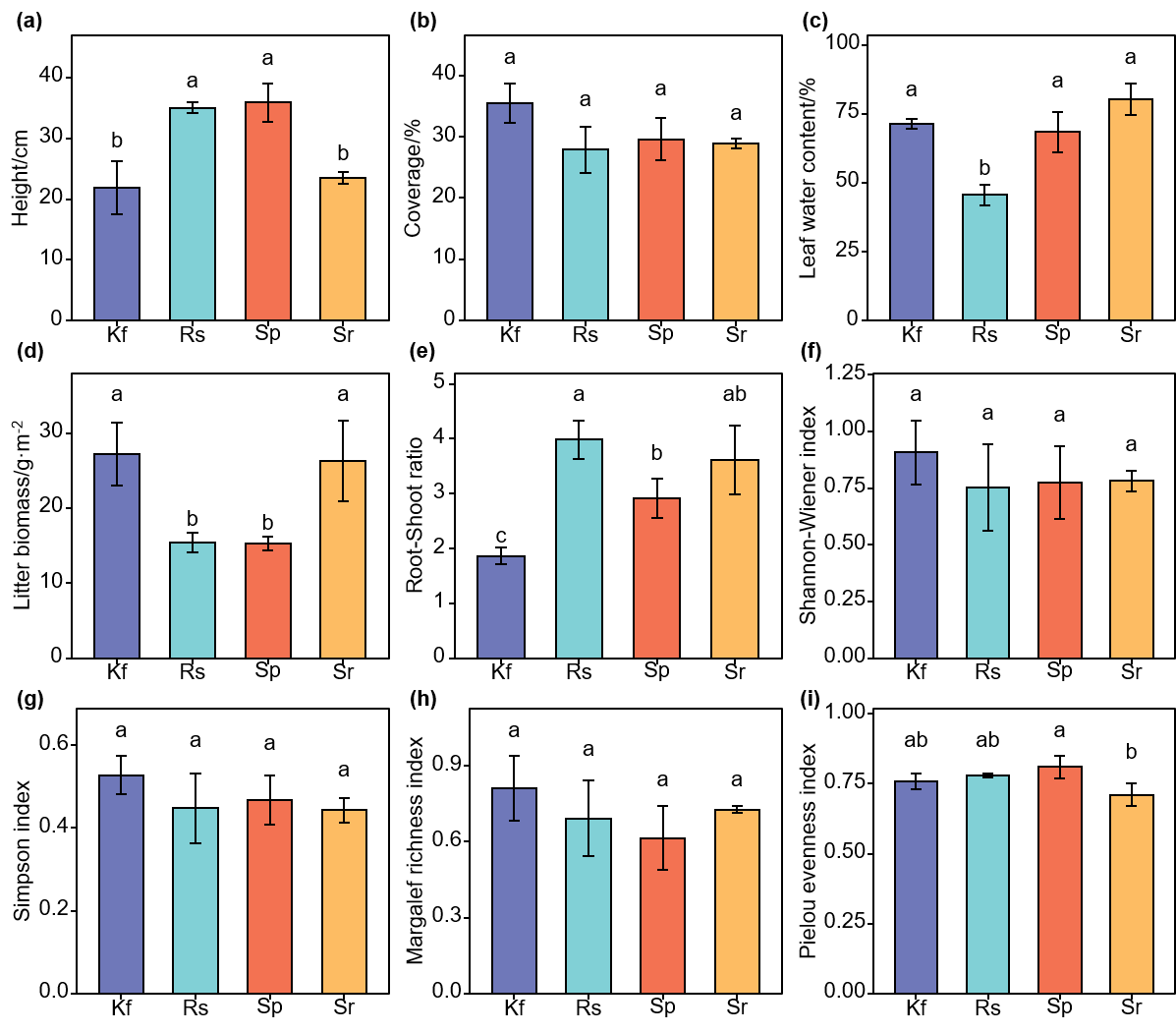


FIGURE S3 Effects of different grassland types on plant community characteristics.

Note: Different lowercase letters indicate significant differences among the four grassland types (*P* < 0.05). Kf: *K. foliatum* type grassland; Rs: *R. soongorica* type grassland; Sp: *S. passerina* type grassland; Sr: *S. regelii* type grassland.


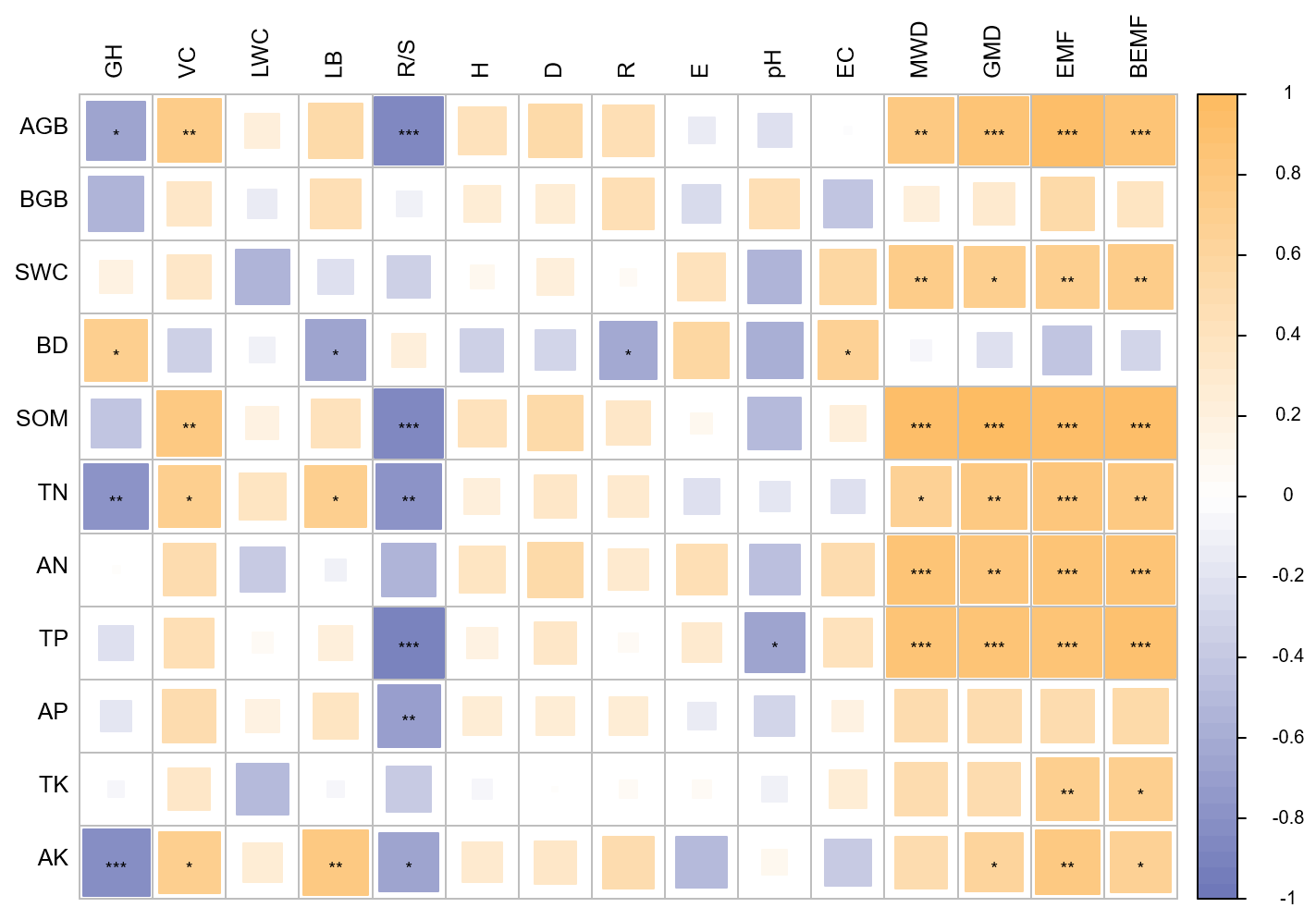


FIGURE S4 Pearson correlation analysis of ecosystem multifunctionality (EMF, BEMF), vegetation characteristics (GH, VC, LWC, LB, R/S), plant diversity (H, D, R, E), soil properties (pH, EC, MWD, GMD) and indicators of ecosystem function (AGB, BGB, SWC, BD, SOM, TN, AN, TP, AP, TK, AK).

Note: EMF: Ecosystem multifunctionality; BEMF: Belowground ecosystem multifunctionality; GH: Height; VC: Coverage; LWC: Leaf water content; LB: Litter bionmass; R/S: Root-Shoot ratio; H: Shannon - Wiener index; D: Simpson index; R: Margalef richness index; E: Pielou evenness index; pH: Soil pH; EC: Soil electrical conductivity; MWD: Mean weight diameter; GMD: Geometric mean diameter; AGB: Aboveground biomass; BGB: Belowground biomass; SWC: Soil water content; BD: Soil bulk density; SOM: Soil organic matter; TN: Soil total nitrogen; AN: Soil alkaline hydrolyzed nitrogen; TP: Soil total phosphorus; AP: Soil available phosphorus; TK: Soil total potassium; AK: Soil available potassium.

*, *p* < 0.05; **, *p* < 0.01; ***, *p* < 0.001.


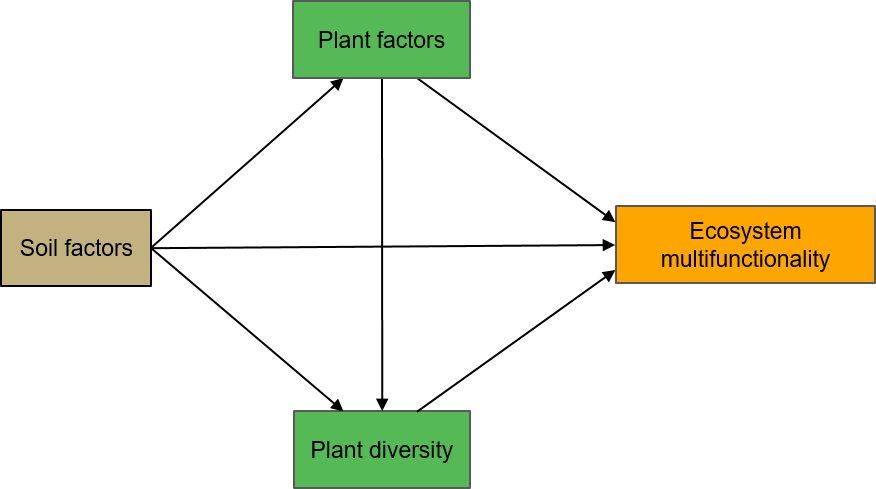


FIGURE S5 A priori structural equation model (SEM) was developed in this study. The model was used to evaluate the effects of soil factors, plant characteristics and plant diversity on ecosystem multifunctionality (EMF).
